# A Comparative Analysis of Patient Profiles and Health Service Utilization between Patent Medicine Vendors and Community Pharmacists in Nigeria

**DOI:** 10.3390/healthcare11182484

**Published:** 2023-09-07

**Authors:** Victor Abiola Adepoju, Olanrewaju Oladimeji

**Affiliations:** 1 Department of HIV and Infectious Diseases, Jhpiego (An Affiliate of Johns Hopkins University), Abuja 190918, Nigeria; 2 Department of Public Health, Faculty of Health Sciences, Walter Sisulu University, Mthatha 5099, Eastern Cape, South Africa; droladfb@gmail.com; 3 Department of Epidemiology and Biostatistics, School of Public Health, Sefako Makgatho Health Sciences University, Pretoria 0208, South Africa

**Keywords:** tuberculosis, patent medicine vendors, drug shops, community pharmacists, service utilization, signs and symptoms

## Abstract

Background: This study examined Nigeria’s socio-demographic profiles and health service utilization patterns of Patent Medicine Vendors (PMVs) and Community Pharmacists (CPs). Method: A cross-sectional study using a structured self-administered questionnaire among 405 retail outlets (322 PMVs and 83 CPs) across 16 Lagos and Kebbi Local Government Areas (LGAs) between June 2020 and December 2020. Results: Results showed that 60.4% were male, 76.3% from Lagos, 58.3% had tertiary education, and 74.1% had medical training. Cough and fever were common symptoms. Significant differences were found in the utilization of STD services (PMVs: 9.2%, CPs: 12.3%, *p* = 0.03), services by age <15 (PMVs: 54.3, CPs: 61.2, *p* < 0.001), and utilization by males (PMVs: 50.8, CPs: 47.1, *p* = 0.013). The study revealed that men visited PMVs more, while CPs used more STI services and childhood visits. Conclusions: The findings suggest that expanding health services among PMVs could target male-dominant diseases, and capacity building of CPs on syndromic STI management could reduce the STI burden.

## 1. Background

The Global Tuberculosis Report released by the World Health Organization stated that about 10 million people were infected with TB in 2019. Nigeria is also one of the three countries that contributed 46% of the missing TB cases, accounting for 11% of missing TB cases that were neither diagnosed nor notified to the National Tuberculosis Program [1]. Although access to care and child survival has improved over the last three decades, many people in LMICs continue to face a variety of structural and social barriers to obtaining formal primary care services, resulting in inequitable access. Since 2006, the World Health Organization has recommended the engagement of pharmacists and drug shops in national TB programs to accelerate TB case finding [2]. Following this, the National TB Program (NTP) in Nigeria has implemented the public/private mix (PPM-DOTS) tuberculosis referral program through the engagement of Patent Medicine Vendors (PMVs), Community Pharmacists (CPs), public and private health facilities that were not previously affiliated with the NTP. PMVs include persons without formal pharmacy training selling orthodox pharmaceutical products on a retail basis and for profit, while CPs formally hold an undergraduate pharmacy degree at the minimum. The Pharmacy Council of Nigeria is responsible for the registration and licensure of all pharmacists and Pharmaceutical Premises, as well as the issuance of permits to pharmacy technicians for the registration and licensure of PMVs [3]. PMVs are licensed to sell approved medicines listed as over-the-counter (OTC) but are forbidden from selling antibiotics and conducting invasive medical procedures. An estimated 200,000 PMVs are operating in Nigeria [4]. They are the first point of care for up to 55% of under-five child illnesses, such as malaria and diarrhea, and 35–55% of adult malaria treatments [5,6,7,8]. They provide initial health services for individuals seeking malaria care in Nigeria. They also remain a major source of self-medication and a ubiquitous component of the informal health system [5]. Well-capacitated PMVs are also pivotal in the provision of quality primary healthcare services, such as the treatment of malaria, diarrhea, and fever [9]. Among the retail vendors, the minority is composed of licensed pharmacies, which are either owned or staffed by formally trained pharmacists and are mainly found in urban centers. In contrast, the majority are informally trained PMVs operating largely in rural areas. While Community Pharmacists are licensed to sell a broader range of drugs, PMVs are limited to only a few approved drugs listed by the Pharmacists Council of Nigeria (PCN). Although both PMVs and CPs operate as vendors, the quality of care differs as Community Pharmacists are perceived to offer higher-quality malaria care services than PMVs [10].

There is a paucity of empirical evidence on the socio-demographics of PMVs and CPs, health service utilization patterns, and how they differ among CPs and PMVs in Nigeria. Given that the scope of practice allowed under the law for these providers could sometimes be restrictive, evidence around utilization patterns of common health conditions and how they vary among providers would help policymakers design a minimum package of care and training needs for case management that these practitioners would require to respond appropriately to the health needs of the community they serve. With a few notable exceptions, little is known about the spectrum of conditions that customers seek treatment for among these providers. The study, therefore, aimed to compare the socio-demographic profile and self-reported health services utilization pattern and common presenting signs and symptoms among PMVs and CPs in Nigeria.

## 2. Methodology

### 2.1. Study Design

A cross-sectional study using structured self-administered questionnaire among 405 retail outlets (322 PMVs and 83 CPs) across 16 Lagos and Kebbi LGAs between June 2020 and December 2020.

### 2.2. Study Setting

The study was conducted in Lagos and Kebbi states, Nigeria, chosen for their urban-rural mix and high TB burden. Sixteen Local Government Areas (LGAs), 13 in urban Lagos and 3 in rural Kebbi were selected. Health services in Nigeria are provided at primary, secondary, and tertiary levels, with Community Pharmacists (CPs) and Patent Medicine Vendors (PMVs) playing a significant role in the TB control program. With Birnin Kebbi as the capital, Kebbi had a population of 352,000 in 2019. Farming is the primary occupation in Kebbi state, and in contrast to Lagos, the majority belong to the lower socio-economic category [11,12]. Lagos is the commercial center and the most populated state in Nigeria, with over 20 million people, 20 Local Government Areas (LGAs), and 57 Local Council Development Areas (LCDA) [13]. 

Recently, the Pharmacists Council of Nigeria adopted the tiered accreditation approach for PMVs in Nigeria. PMVs are categorized into three tiers based on their health qualifications in this arrangement. This system recognizes the background educational qualification(s) that PMVs have. Each tier was authorized to practice service provision consistent with their qualifications. Tier one includes PMVs lacking health qualifications but can read and write. Tier two are health-qualified PMVs who possess health-related degrees as recognized by the Pharmacists Council of Nigeria (e.g., Nurses, community health extension workers, and midwives), while tier three are qualified pharmacy technicians. Each of the tiers has a scope of practice as stipulated by the Pharmacists Council of Nigeria.

### 2.3. Study Size

A total of 405 PMVs and CPs were included in the study. We calculated sample size using the formula n = a^2^b/d^2^, where n = sample size, a = Z statistic for a level of confidence, b = prevalence, and d = precision or confidence interval. The level of confidence of 95% is conventional, at which the value for a is 1.96 and d is 0.05. A recent patient pathway study found that 27% of people with TB first sought medical care from private chemist shops before any other formal or informal provider [14]. This result equates to a ‘b’ value of 0.27, giving an approximate sample size of 353 participants. With an assumption of a 20% non-response rate, a total of 405 participants were invited to participate in the study.

### 2.4. Sampling Technique

A 4-stage sampling technique (summarized below) was used to select participants for this study.

#### 2.4.1. Stage 1: Selection of Study LGAs

A total of 16 LGAs (13 LGAs from Lagos and 3 LGAs from Kebbi) were purposively selected. These LGAs have a high TB burden and retail store presence in these states.

#### 2.4.2. Stage 2: Selection of PMVs and CPs

The list of CPs and PMVs registered in each state as of 2019 was requested from professional associations and stratified into those formally affiliated and not affiliated with the NTP. The investigators then filtered and compared this list with that of NTP, filtering out PMVs and CPs already affiliated with NTP from the universal list.

#### 2.4.3. Stage 3 Selection of PMVs and CPs Not Formally Affiliated with NTBLCP

The required number of participating non-NTP affiliated CPs and PMVs was systematically sampled in each of the 16 LGAs, proportionate to the number of PMVs and CPs in the LGA until the sample size was reached.

#### 2.4.4. Stage 4: Selection of PMV and CP Shop Owners

The purposive sampling technique was used in selecting the required numbers of CPs and PMVs (shop owners) interviewed in the selected premises. A purposive sample of 322 PMVs and 83 community pharmacy store owners were recruited for the study. The chief pharmacist/PMV was interviewed for the CPs and PMVs. Where the Chief Pharmacist or PMV was not available, they were replaced by another attending pharmacist for CP or a PMV assistant who met the inclusion criteria. If more than one CP/PMV met the inclusion criteria, simple random balloting was used to select respondents to be interviewed.

### 2.5. Participants

Study population included 405 CPs and PMVs (owners of retail outlets) in Lagos and Kebbi states.

### 2.6. Inclusion Criteria

- Age ≥ 18 years;
- CPs and PMVs registered with relevant professional associations;
- PMVs and CPs not currently affiliated with NTBLCP;
- PMVs and CPs currently licensed by Pharmacy Council of Nigeria;
- Having a physical shop where the vendor operates.

We excluded any CP or PMV who did not meet the inclusion criteria.

### 2.7. Variables

The outcome/dependent variable was the ‘type of provider’, categorized into ‘PMV or CP’. Independent variables include participants’ gender, LGA, type of business (PMV/CP), highest educational qualification, medical education, year of practice, number of apprentices, clients seen per day, PMV accreditation tier, practice setting, qualification, previous TB training, number of post-graduation years of experience and awareness of DOTS and DOTS facilities. Study variables were presented using descriptive statistics such as frequency and percentage. Descriptive statistics, including frequency distributions, means, and standard deviations, were used to describe respondents’ socio-economic and demographic characteristics. Independent *t*-test statistic was used to test for differences in service utilization by type of sign/symptoms, age, gender, and purchaser and how these differ between PMVs and CPs.

### 2.8. Data Source and Measures

The questionnaire administered for this study contains 15 questions divided into 2 sections.

Section 1 contains 12 questions about the socio-demographic information such as sex, LGA, type of business (PMV/CP), highest educational qualification (primary, secondary, tertiary, and none), medical education (Y/N), year of practice (0–5, 6, or more), prior TB training (Y/N), number of apprentices (0, 1–2, 3 or more), clients seen per day (1–10, 11–20, 20, or more), and PMV accreditation tier (1, 2, or 3).

Section 2 assesses provider-reported service utilization and common presenting clinical signs and symptoms demographics. In Q1, providers were asked to estimate the proportion of their visiting clients with cough, fever, diarrhea, STI, and family planning needs for every 10 patients visiting their premises over the last 3 months. In Q2, providers also estimated the number of adults and children for every 10 patients who visited their premises in the last 3 months. In Q3, participants estimated the proportion of clients visiting their premises over the last 3 months who bought medication for themselves and someone sick at home, while in Q4, providers estimated the proportion of clients visiting their premises over the last 3 months who were adults >15 years old or child ≤15 years of age.

### 2.9. Bias

In order to minimize bias, the study comprises private non-NTP affiliated retail vendors across 16 LGAs from both urban and rural populations in northern and southern Nigeria, as well as large and small-sized CPs and PMVs.

### 2.10. Data Collection

The researchers received clearance from relevant professional associations to gain legitimacy since PMVs may perceive strangers as government monitoring agents. The Chair of the associations introduced researcher to their members and explained the goal of the study. A total of 16 research assistants (fluent in both English and local languages, one per LGA) received 3-day training on data collection processes, survey protocol, standard operating procedure, Excel-based data collection template, and Global Positioning Systems (GPS) to capture geospatial data of CPs and PMVs. The study was piloted among 10 CPs and PMVs in 2 LGAs prior to the data collection to test the knowledge of the trained data collectors. The pilot study took place outside the sampling areas in Kebbi and Lagos states, and based on the information generated, further adjustments were made to the wording of the questions to make it easily understood before adoption in the main study. Questions were self-administered following extensive explanation and clarifications. Returned questions were read by research assistants. Study objective was masked from data collectors, and daily data reviews were held to assess data completion, missing information, and double counting. The questionnaire was divided into two sections with a total of 15 questions, which sought information on participants’ socio-demographics (10) and assessed service utilization and commonly presenting clinical signs and symptoms among PMVs and CPs (5). There were 4 supervisors, and each managed a group of 4 research assistants. The supervisors randomly selected sample data collected, reviewed, approved, and ensured interrater reliability. Informed consent was taken from each eligible participant prior to the administration of the questionnaire. When the shop owner was not around during the visit, research assistants rescheduled a convenient time. During the revisit, when the Chief Pharmacist or PMV owner was still not available, they were replaced by another attending pharmacist for CP or a PMV assistant who met the inclusion criteria. If more than one CP/PMV met the inclusion criteria, simple random balloting was then used to select interview respondents. Data were collected between June and December 2020.

### 2.11. Data Analysis

Responses were first entered into Microsoft Excel, then checked, cleaned, and imported into Statistical Software for Social Science (SPSS) version 17 for coding, categorization, and statistical analyses. In addressing the research objective, we applied descriptive statistics (percentages and numbers) to summarize the categorical variables:socio-demographic variables and median (%, proportion) estimate of service utilization and commonly presenting signs and symptoms, age, and purchasing pattern as self-reported by PMVs versus CPs. Descriptive statistics, including frequency distributions, means, and standard deviations, were used to describe socio-economic and demographic characteristics of the respondents. Independent *t*-test statistic was used to test for differences in service utilization by type of sign/symptoms (cough, fever, diarrhea, contraceptive, and STD), by age (<15 and >15 years), by gender (male versus female) and by purchaser (self or sick at home) and how these differ between the PMVs and CPs.

## 3. Results

In Table 1, the majority 243 (60.4%) of participants were male, and 309 (76.3%) of them were from the state of Lagos, 236 (58.3%) had the tertiary educational qualification, 300 (74.1%) had medical education, 290 (71.6%) spent 6 years or more in practice, 286 (70.6%) were not previously trained on TB, 151 (37.3%) had one to two apprentices, 255 (63%) consulted over 20 customers/day, and 262 (82.5%) belonged to PMV tier 1 accreditation.

Table 2 reveals that overall, there is no significant difference in mean service utilization for cough, fever, diarrhea, contraceptive services, and by type of purchaser of service (purchasing for self or someone sick at home) among PMVs and CPs. There was a significant difference in the utilization of STD services, utilization by age, and gender between the PMVs and CPs.

**Figure 1:**
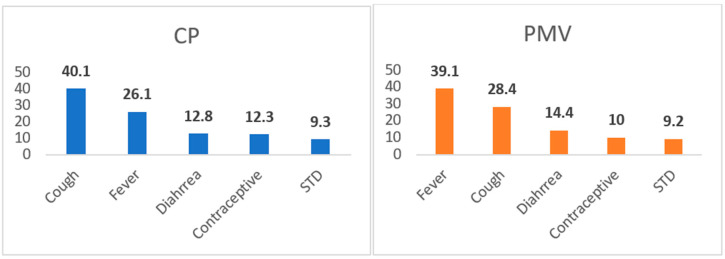
Common presenting signs and symptoms among PMVs and CPs in Nigeria.

In Figure 1, cough and fever were the most common symptoms presented to CPs and PMVs, respectively.

## 4. Discussion

The objective was to compare the socio-demographic profile and self-reported health services utilization pattern and common presenting signs and symptoms among PMVs and CPs in Nigeria. The study showed that cough (28.4–40.1%) and fever (26.1–34.1%) were, respectively, the most common services utilized among PMVs and CPs in Nigeria. Also, the investigators observed a significantly high mean utilization of STI services among CPs when compared to PMVs. Furthermore, a significantly greater service utilization was observed among men and children <15 years who visited PMV and CP, respectively. These findings are relevant for the engagement of PMVs and CPs in Nigeria in disease detection and capacity building, as CPs and PMVs remain the first port of call for the majority of the initial health-seeking volume in Nigeria. Notably, the two settings have similar rates of visits for STI, differing by only one-tenth of a percent. This situation could be explained by the higher male presence in the setting with the onsite antibiotics, as women are less overtly symptomatic for some strains. Similarly, a study from Kogi and Kwara states in Nigeria reported that headache, fever, and cough accounted for over 70% of services utilized among PMVs [15]. In southeast Nigeria, antimalaria services accounted for 95% of services used by nursing mothers and young people [16], while in another study in Nigeria, cough and malaria were highlighted as leading presenting ailments among PMVs and CPs, followed by STI [17]. Given that malaria, pneumonia, diarrhea, and respiratory infections are the leading causes of under-five deaths in Nigeria, engaging PMVs and CPs to identify, refer, or treat these common conditions in line with national policy and guidelines and, based on these utilization patterns, is urgently needed.

The study also reported a significantly higher mean utilization of STI services among CPs compared to PMVs, which is quite instructive. This finding could be the result of policy that forbids PMVs from stocking prescription-only drugs, such as antibiotics needed to treat STIs. Another explanation could be that CPs serve the sexual and reproductive health needs of young people and provide health advisory and post-exposure risk reduction counseling, unlike PMVs, who only sell and dispense requested medications [18,19]. The differential in utilization for STI might be due to prescription access among CPs and PMVs in Nigeria. These findings were similarly echoed by a qualitative exploratory study in Nigeria, although the study failed to differentiate utilization patterns between PMVs and CPs [19]. CPs play a strategic role in managing STI, especially for young people, who are more comfortable engaging CPs for STI services than staff of traditional health facilities. This preference helps them to avoid stigma, societal criticism, and judgmental health service delivery. Community Pharmacists also reportedly manage more STIs than government health institutions in Ghana [20], hence the need to integrate them into STI surveillance and build their capacity in diagnosing and reporting syndromic STI management through their inclusion in primary healthcare (PHC) systems. Their continued exclusion despite the high STI service utilization in their premises will only perpetuate the vicious cycle of STI transmission and reinfection in Nigeria. The study did not specifically address female patient visits for STIs and the issues associated with meeting their needs, especially those with asymptomatic infection, which is an important area for future research.

Another interesting finding from this study is the significantly higher mean service utilization among children visiting CPs compared to PMVs. According to the 2018 Nigeria Demographic and Health Survey (NDHS), the under-five mortality rate in Nigeria was 132 per 1000 live births, an increase from 128 per 1000 live births in 2013 [21]. More than 75% of these deaths resulted from malaria, pneumonia, and diarrhea [22]. That notwithstanding, the knowledge needed to manage these childhood conditions properly remains poor among these providers in Nigeria [23,24].

This study showed a significantly higher mean proportion of men who utilized health services among PMVs than CPs. The demographic pattern of some diseases, such as tuberculosis, showed that TB is more prevalent globally among men than women [25] and that Nigerian men have twice as much TB as women but are less likely to use public health services [26,27,28]. A previous study in Kenya reported that pharmacies and/or drug stores are preferred options for men to access HIV Self-Test kits [29]. Therefore, taking PMV engagement to scale by the government has the potential advantage of improving access and utilization of health services for male-dominant diseases. Despite the novel findings of the study, it has some limitations that should be considered in interpreting the results. Due to the lack of sales records, we used the “last 10 visits” self-reported by interviewees (PMV and CP) as a proxy for visit volume. Also, the study could have been strengthened by in-depth interviews with participants from both settings, which was not feasible due to the scope and design of our study.

## 5. Conclusions

This study has further advanced the body of knowledge on how service utilization among CPs and PMVs differ by gender, age, and disease type in Nigeria. The findings could contribute to improvement in finding and treating common illnesses in the community at the point of initial care-seeking. Expanding health services among PMVs will help to address certain male-predominant diseases. At the same time, capacity building of CPs on childhood illnesses and syndromic STI management could reduce the disease burden because of the magnitude of the demand and utilization of these diseases and because of their age and gender compositions among end-users visiting these providers.

## Figures and Tables

**Table 1:** Socio-demographic characteristics.

Variables	PMV	CP	Total
Sex			
Male	186 (58.3%)	57 (68.7%)	243 (60.4%)
Female	133 (41.7%)	26 (31.3%)	159 (39.6%)
State			
Lagos	230 (71.4%)	79 (95.2%)	309 (76.3%)
Kebbi	92 (28.6%)	4 (4.8%)	96 (23.7%)
LGA			
Agege	29 (9.0%)	8 (9.6%)	37 (9.1%)
Ikeja	3 (0.9%)	2 (2.4%)	5 (1.2%)
Ifelodun Ajeromi	16 (5.0%)	5 (6.0%)	21 (5.2%)
Alimosho	64 (19.9%)	29 (34.9%)	93 (23.0%)
Kosofe	7 (2.2%)	10 (12.0%)	17 (4.2%)
Shomolu	0 (0.0%)	3 (3.6%)	3 (0.7%)
Ojo	51 (15.8%)	13 (15.7%)	64 (15.8%)
Badagry	6 (1.9%)	0 (0.0%)	6 (1.5%)
Apapa	28 (8.7%)	0 (0.0%)	28 (6.9%)
Mushin	8 (2.5%)	1 (1.2%)	9 (2.2%)
Amuwo odofin	5 (1.6%)	6 (7.2%)	11 (2.7%)
Oshodi	3 (0.9%)	2 (2.4%)	5 (1.2%)
Ifako Ijaye	10 (3.1%)	0 (0.0%)	10 (2.5%)
Koko-besse	35 (10.9%)	1 (1.2%)	36 (8.9%)
Argungu	50 (15.5%)	0 (0.0%)	50 (12.3%)
Yauri	7 (2.2%)	3 (3.6%)	10 (2.5%)
Level of Education			
Primary	7 (2.2%)	0 (0.0%)	7 (1.7%)
Secondary	162 (50.3%)	0 (0.0%)	162 (40.0%)
Tertiary	153 (47.5%)	83 (100.0%)	236 (58.3%)
Medical Education			
No	105 (32.6%)	0 (0.0%)	105 (25.9%)
Yes	217 (67.4%)	83 (100%)	300 (74.1%)
Specify Medical Education (N = 201)			
Tier 1 PMV	262 (82.6%)	N/A	262 (82.6%)
Tier 2 PMV	49 (15.5%)		49 (15.5%)
Tier 3 PMV	6 (1.9%)		6 (1.9%)
Years in Business			
0–5 yrs	88 (27.3%)	27 (32.5%)	115 (28.4%)
6 yrs and above	234 (72.7%)	56 (67.5%)	290 (71.6%)
Trained on TB			
No	245 (76.1%)	41 (49.4%)	286 (70.6%)
Yes	77 (23.9%)	42 (50.6%)	119 (29.4%)
Number of Apprentices			
None	129 (40.1%)	1 (1.2%)	130 (32.1%)
1–2	132 (41.0%)	19 (22.9%)	151 (37.3%)
3 and above	61 (18.9%)	63 (75.9%)	124 (30.6%)
Clients per actual average no. (N = 209)			
1–10	110 (58.2%)	5 (25.0%)	115 (55.0%)
11–20	79 (41.8%)	15 (75.0%)	94 (45.0%)
Keep any treatment record			
No	234 (72.7%)	43 (51.8%)	277 (68.4%)
Yes/not seen	52 (16.1%)	20 (24,1%)	72 (17.8%)
Yes/seen	36 (11.2%)	20 (24.1%)	56 (13.8%)
Referred coughing person in one month			
No	164 (50.9%)	45 (54.2%)	209 (51.6%)
Yes	158 (49.1%)	38 (45.8%)	196 (48.4%)
Kept referral records			
No	276 (85.7%)	67 (80.7%)	343 (84.7%)
Yes/not seen	30 (9.3%)	9 (10.8%)	39 (9.6%)
Yes/seen	16 (5.0%)	7 (8.4%)	23 (5.7%)
Willing to be engaged as a TB treatment center			
No	8 (2.5%)	1 (1.2%)	9 (2.2%)
Yes	314 (97.5%)	82 (98.8%)	396 (97.8%)

**Table 2:** Pattern of self-reported utilization of health services among PMVs and CPs in Nigeria.

Variables	PMV	CP	*p*-Value
**Types of health services utilized**			
Out of 10 visiting clients, what proportion are with cough (N = 396)	28.4 ± 10.71	26.1 ± 7.97	0.065
Out of 10 visiting clients, what proportion are with fever (N = 395)	39.1 ± 14.63	40.1 ± 11.91	0.514
Out of 10 visiting clients, how many are with diarrhea (N = 391)	14.4 ± 8.98	12.8 ± 7.08	0.083
Out of 10 visiting clients, what proportion are with family planning needs (N = 387)	10 ± 8.59	9.3 ± 5.65	0.333
Out of 10 visiting clients, what proportion are with STDs (N = 386)	9.2 ± 8.74	12.3 ± 7.12	*0.03
**Utilization by Age**			
Out of 10 visiting clients, what proportion are usually adults <15 yrs (N = 398)	54.3 ± 17.77	61.2 ± 15.01	* <0.001
Out of 10 visiting clients, what proportion are usually children >15 yrs (N = 398)	45.7 ± 17.51	38.6 ± 14.83	
**Utilization by type of purchaser**			
Out of 10 visiting clients, what proportion usually buy for himself/herself (N = 399)	61.9 ± 16.68	63.3 ± 13.44	0.429
Out of 10 visiting clients, what proportion usually buy for someone who is sick at home (N = 400)	38.1 ± 17.17	36.6 ± 13.46	0.364
**Utilization by Gender**			
Out of 10 visiting clients, what proportion are usually male clients (N = 399)	50.8 ± 17.29	47.1 ± 9.94	*0.013
Out of 10 visiting clients, what proportion are usually female clients (N = 400)	49.2 ± 17.15	52.9 ± 9.94	

* Significant *p*-value < 0.05.

## Data Availability

The data presented in this study are available on request from the corresponding author. The data are not publicly available due to privacy restrictions.
